# On the Stochastic Dynamics for the Regularized Kappa-Distributed Plasmas

**DOI:** 10.3390/e27111138

**Published:** 2025-11-05

**Authors:** Ran Guo

**Affiliations:** Department of Physics, College of Science, Civil Aviation University of China, Tianjin 300300, China; rguo@cauc.edu.cn

**Keywords:** regularized Kappa distribution, generalized fluctuation-dissipation relation, Fokker–Planck equation, Langevin equation

## Abstract

The generalized fluctuation–dissipation relations that produce the regularized Kappa distributions are studied. The two-variable Fokker–Planck equation, as well as its reductions in the absence of potential and in the overdamped limit, are considered. All these Fokker–Planck equations have the regularized Kappa distributions as the stationary solutions if the friction and diffusion coefficients satisfy the generalized fluctuation–dissipation relations. In addition, we prove that the principle of detailed balance holds for all the stationary solutions derived in this work.

## 1. Introduction

The supra-thermal particles are widely observed in space plasmas. In early studies, Olbert [1], as well as Vasyliunas [2], proposed the three-dimensional standard Kappa distribution (SKD) to model the supra-thermal electrons in the magnetosphere.

(1) $$f_{SKD}\left(v\right)=\frac{n_{0}}{{\left(\kappa \pi \theta^{2}\right)}^{3/2}}\frac{\Gamma (\kappa +1)}{\Gamma (\kappa -\frac{1}{2})}{\left(1+\frac{v^{2}}{\kappa \theta^{2}}\right)}^{-\kappa -1},$$

where $n_{0}$ is the number density, κ is the parameter measuring the deviation from the Maxwellian distribution, and θ is the thermal speed. After that, numerous studies found that the SKD is a successful model to describe the non-Maxwellian particles in various plasma environments, such as solar winds [3,4,5], the planetary magnetosphere [6,7], and the Earth’s magnetotail [8]. These Kappa-distributed particles can significantly affect the behaviors of plasmas, such as the collision frequencies [9,10], the transport coefficients [11,12,13], the thermodynamic properties [14,15], and the electrostatic and electromagnetic waves [16,17,18,19,20,21,22,23].

Although the SKD is successfully applied to many plasma systems, it has some unexpected characteristics. The most notable one is that the *l*-order velocity moment of SKD (1) diverges when l≥2κ+1 [24]. As a result, a complete fluid theory involving higher-order moments is difficult to establish. To overcome the difficulty, Scherer et al. [24] proposed the three-dimensional regularized Kappa distribution (RKD):

(2) $$f_{RKD}\left(v\right)=\frac{n_{0}}{{\left(\pi \kappa \theta^{2}\right)}^{3/2}}\frac{1}{U\left(\frac{3}{2},\frac{3}{2}-\kappa ,\alpha^{2}\kappa \right)}{\left(1+\frac{v^{2}}{\kappa \theta^{2}}\right)}^{-\kappa -1}\exp\left(-\alpha^{2}\frac{v^{2}}{\theta^{2}}\right),$$

where U(a,b,z) is the Kummer U function, and α is the cut-off parameter. The RKD could be identified as the SKD multiplied by an exponential cut-off factor $\exp(-\alpha^{2}v^{2}/\theta^{2})$. The regularized distribution (2) recovers the SKD (1) in the limit of α→0, and the Maxwellian distribution in the limit of κ→∞ and α→0. The velocity moments of RKD are finite for arbitrary order with a non-zero cut-off parameter α [24,25]. In addition, the allowable range of the kappa parameter is extended to κ>0 for RKD, while it is κ>3/2 for SKD to ensure the finite kinetic temperature (which is related to the second-order velocity moment) [24].

The RKD has been used to study various properties of space plasmas. Fichtner et al. studied the entropy of a spatially homogeneous plasma following the RKD [26]. Husidic et al. [27] solved the linear dispersion relation of parallel electromagnetic modes in the regularized Kappa-distributed plasmas, and found that the dispersion curves of the RKD are almost identical to those of the SKD when α is small enough. Both linear and nonlinear ion-acoustic waves [28,29] were studied in regularized Kappa-distributed plasmas, showing the existence of the wave for 0<κ<3/2, which is unavailable for the SKD. Gaelzer and Ziebell [30] investigated the effects of the RKD on the collisional charging of dust grains. Their results indicate that the difference between the cut-off parameters of electrons and ions plays an important role in the dust charging process. The temperature anisotropy instability was analyzed in the plasmas where the supra-thermal halo electrons are described by the RKD and the quasi-thermal core electrons by the Maxwellian distribution [31]. It is found that the instability is significantly enhanced in the range of κ<3/2 that is not accessible for the SKD. The RKD was also used in the kinetic exospheric model of the solar wind to compare the theoretical results with the observations [32].

There are several physical mechanisms explaining the supra-thermalization of the distribution [33,34,35,36,37,38,39,40,41,42,43,44,45]. Among these mechanisms, one class of explanations is based on solving the Fokker–Planck (FP) equation. Hasegawa et al. proved that [33] the SKD could be a stationary solution of the FP equation describing the plasma immersed in a super-thermal radiation field. Biró and Jakovác [46] considered the one-dimensional FP equation with multiplicative noise and obtained the stationary distribution with the power-law form. Du [41] derived the SKD as the steady-state solution from the two-variable Langevin equation and its associated FP equation if the friction and diffusion coefficients follow a generalized fluctuation–dissipation relation (GFDR). Oylukan and Shizgal [47] explored a series of FP equations and found that the SKD is only one of the steady-state solutions. Nevertheless, the physical interpretation of the cut-off factor for RKD is still unclear. Therefore, the present work aims to investigate the stochastic process that produces a stationary RKD by solving the associated FP equation.

The paper is organized as follows. In Section 2, we derive several RKDs as the stationary solutions under the assumptions of the GFDR in different cases. In Section 3, the principle of detailed balance is tested for all the stationary solutions derived in Section 2. At last, we draw the conclusions in Section 4.

## 2. The Regularized Kappa Distribution as a Stationary State Under the Generalized Fluctuation–Dissipation Relation

As is well known, the Brownian particle in the potential is modeled by the two-variable Langevin equation [48]

(3) $$\frac{dx}{dt}=\frac{p}{m},\;\frac{dp}{dt}=-\frac{dV(x)}{dx}-\gamma \frac{p}{m}+f\left(x,p,t\right),$$

where V(x) is the potential energy, γ is the friction coefficient, and f(x,p,t) is the random force satisfying

(4) $$\langle f(x,p,t)\rangle =0,\;\left\langle f\left(x,p,t\right)f\left(x,p,t^{\prime }\right)\right\rangle =2D\delta \left(t-t^{\prime }\right).$$

We assume both the friction and diffusion coefficients are functions of (x,p), i.e.,

(5) γ=γ(x,p),D=D(x,p),

which may be reasonable in active systems, dusty plasmas, and tokamak plasmas [49,50,51]. Due to the (x,p)-dependent coefficients, the resulting FP equation is different when we choose different stochastic integral rules, such as Ito’s, Stratonovich’s, and backward-Ito’s rules. Each of them has advantages and disadvantages, and they can be converted into one another through specific transformations [52]. In this work, we adopt the backward-Ito’s rule of the stochastic integral, which may lead to an elegant form of the GFDR [48,52]. Therefore, the associated FP equation could be written as [48,52]

(6) $$\frac{\partial \rho }{\partial t}=-\frac{p}{m}\frac{\partial \rho }{\partial x}+\frac{\partial }{\partial p}\left[\frac{dV(x)}{dx}+\gamma \left(x,p\right)\frac{p}{m}\right]\rho +\frac{\partial }{\partial p}\left[D(x,p)\frac{\partial }{\partial p}\right]\rho ,$$

where ρ=ρ(x,p,t) is the distribution function of the stochastic process. The stationary solution of the FP Equation (6) is given by [41]

(7) $$\rho_{s}=\rho_{s}\left(E\right)=\frac{1}{Z}\exp\left(-\int \frac{\gamma }{D}E\right),$$

where *Z* is the normalization constant and $E=p^{2}/\left(2m\right)+V\left(x\right)$ is the total energy of the Brownian particle. It should be noted that the friction coefficient defined in the present work slightly differs from the one in [41], resulting in a different form of the stationary solution. If the friction and diffusion coefficients satisfy the GFDR,

(8) $$\frac{\gamma }{D}=\frac{\beta }{1-\kappa^{*}\beta E},$$

the stationary solution has a power-law form [41]

(9) $$\rho_{s}\left(E\right)=\frac{1}{Z_{SKD}}{\left(1-\kappa^{*}\beta E\right)}_{+}^{\frac{1}{\kappa^{*}}}.$$

Here, $\beta =1/(k_{B}T)$ is the inverse temperature and ${\left(x\right)}_{+}=x$ for x≥0 and zero otherwise. Through parameter transformation $\kappa =-1/\kappa^{*}$, the GFDR (8) becomes

(10) $$\frac{\gamma }{D}=\frac{\beta }{1+\beta E/\kappa },$$

and the solution (9) can be written as

(11) $$\rho_{s}\left(E\right)=\frac{1}{Z_{SKD}}{\left(1+\frac{\beta E}{\kappa }\right)}_{+}^{-\kappa },$$

which is exactly the one-dimensional SKD [18,53] derived by integrating Equation (1). For $\kappa^{*}=0$ or equivalently κ→∞, the GFDR (8) recovers the standard fluctuation–dissipation relation (SFDR) γ/D=β, leading to a Maxwellian distribution $\rho_{s}=\exp\left(-\beta E\right)/Z_{M}$ for the equilibrium state.

If it is further assumed that

(12) $$\frac{\gamma }{D}=\frac{\beta }{1+\beta E/\kappa }+\beta \alpha^{2},$$

one derives the stationary solution (7) of the FP equation

(13) $$\rho_{s}\left(E\right)=\frac{1}{Z_{RKD}}{\left(1+\frac{\beta E}{\kappa }\right)}_{+}^{-\kappa }\exp\left(-\alpha^{2}\beta E\right),$$

where $Z_{RKD}=\int \int {\left(1+\beta E/\kappa \right)}_{+}^{-\kappa }\exp\left(-\alpha^{2}\beta E\right)dxdp$. Equation (13) could be identified as the regularization of the one-dimensional SKD (11). It is worth noting that Equation (13) differs from the one-dimensional RKD in Ref. [54], which is obtained by integrating the three-dimensional RKD (2). Comparison of the two kinds of one-dimensional RKD can be found in Appendix A.

The GFDR (12) clarifies how the power-law and exponential terms arise in the RKD. The first term on the right-hand side of Equation (12) is the origin of the SKD, while the second term introduces the exponential regularization. A possible construction satisfying the GFDR (12) is that the friction and diffusion coefficients are both linear functions of the energy. Without loss of generality, one can write

(14) $$\begin{array}{cc} \gamma (E)= & \gamma \left(0\right)\left(1+\alpha^{2}+\alpha^{2}\frac{\beta E}{\kappa }\right), \end{array}$$

(15) $$\begin{array}{cc} D(E)= & D\left(0\right)\left(1+\frac{\beta E}{\kappa }\right). \end{array}$$

Considering that γ and *D* should be constant when the system is in equilibrium, we have γ(0)/D(0)=β, leading to the GFDR (12).

If the system is uniform ∂ρ/∂x=0 and no potential is present V(x)=0, the FP Equation (6) reduces to

(16) $$\frac{\partial \rho }{\partial t}=\frac{\partial }{\partial p}\left[\gamma \left(p\right)\frac{p}{m}\rho \right]+\frac{\partial }{\partial p}\left[D(p)\frac{\partial }{\partial p}\rho \right],$$

which describes a diffusion process in the momentum space. The friction (14) and the diffusion coefficient (15) become

(17) $$\begin{array}{cc} \gamma (p)= & \gamma \left(0\right)\left(1+\alpha^{2}+\alpha^{2}\frac{\beta }{\kappa }\frac{p^{2}}{2m}\right), \end{array}$$

(18) $$\begin{array}{cc} D(p)= & D\left(0\right)\left(1+\frac{\beta }{\kappa }\frac{p^{2}}{2m}\right). \end{array}$$

Consequently, the steady-state solution of Equation (16) is a velocity RKD

(19) $$\rho_{s}\left(v\right)=\frac{1}{Z_{RKD}}{\left(1+\frac{1}{\kappa }\frac{v^{2}}{\theta^{2}}\right)}_{+}^{-\kappa }\exp\left(-\alpha^{2}\frac{v^{2}}{\theta^{2}}\right),$$

where $\theta =\sqrt{2/(m\beta )}$ is the thermal speed. It should be noted that although the Pearson type IV distribution in statistics is also the product of the power-law and the exponential part [55],

(20) $$\rho \left(x\right)\propto {\left(1+\frac{x^{2}}{a^{2}}\right)}^{-m}\exp\left[-\nu \arctan\left(\frac{x}{a}\right)\right],$$

the RKD (19) has a different decay in the exponent.

If the Brownian particle is in a strong frictional medium, the Langevin Equation (3) approximates to [48]

(21) $$\frac{dx}{dt}=-\frac{1}{\gamma }\frac{dV(x)}{dx}+\frac{1}{\gamma }f\left(x,t\right)$$

with the noise satisfying

(22) $$\langle f(x,t)\rangle =0,\;\left\langle f\left(x,t\right)f\left(x,t^{\prime }\right)\right\rangle =2D\left(x\right)\delta \left(t-t^{\prime }\right).$$

Therefore, the overdamped FP equation (also called the Smoluchowski equation) under the backward Ito’s stochastic integral rule is

(23) $$\frac{\partial \rho }{\partial t}=\frac{\partial }{\partial x}\left[\frac{1}{\gamma (x)}\frac{dV(x)}{dx}\rho \right]+\frac{\partial }{\partial x}\left[\frac{D(x)}{\gamma^{2}\left(x\right)}\frac{\partial }{\partial x}\rho \right],$$

and its stationary solution is given by [41]

(24) $$\rho_{s}\left(x\right)=\frac{1}{Z}\exp\left(-\int \frac{\gamma }{D}V\right).$$

If the GFDR is assumed to be

(25) $$\frac{\gamma }{D}=\frac{\beta }{1+\beta V(x)/\kappa }+\beta \alpha^{2},$$

one derives the stationary solution

(26) $$\rho_{s}\left(x\right)=\frac{1}{Z_{RKD}}{\left[1+\frac{\beta V(x)}{\kappa }\right]}_{+}^{-\kappa }\exp\left[-\alpha^{2}\beta V\left(x\right)\right].$$

## 3. The Detailed Balance

Once the stationary solution of the FP equation is derived, we can judge whether the principle of detailed balance holds for the solution. The sufficient and necessary conditions for the detailed balance can be expressed by the friction and diffusion coefficients of the FP equation [56]. For a physical system described by a set of variables $q={\left\{q_{i}\right\}}_{i=1,\ldots ,N}$, we denote the time-reversal variables as $\tilde{q}={\left\{\epsilon_{i}q_{i}\right\}}_{i=1,\ldots ,N}$, where $\epsilon_{i}=\pm 1$ depends on whether $q_{i}$ changes its sign through the time-reversal transformation. For a general FP equation,

(27) $$\frac{\partial \rho (q,t)}{\partial t}=-\sum_{i}\frac{\partial }{\partial q_{i}}A_{i}\left(q\right)\rho \left(q,t\right)+\frac{1}{2}\sum_{i,k}\frac{\partial^{2}}{\partial q_{i}\partial q_{k}}B_{ik}\left(q\right)\rho \left(q,t\right),$$

with a formal stationary solution

(28) $$\rho_{s}=\frac{1}{Z}\exp\left[-\phi \left(q\right)\right],$$

the sufficient and necessary conditions of the detailed balance could be written as [57]

(29) $$B_{ik}\left(q\right)=\epsilon_{i}\epsilon_{k}B_{ik}\left(\tilde{q}\right),$$

(30) $$Q_{i}-\frac{1}{2}\sum_{k}\frac{\partial B_{ik}}{\partial q_{k}}=-\frac{1}{2}\sum_{k}B_{ik}\frac{\partial \phi }{\partial q_{k}},$$

(31) $$\sum_{i}\left(\frac{\partial J_{i}}{\partial q_{i}}-J_{i}\frac{\partial \phi }{\partial q_{i}}\right)=0,$$

where the irreversible and reversible drift coefficients are defined as, respectively,

(32) $$Q_{i}\left(q\right)=\frac{1}{2}\left[A_{i}\left(q\right)+\epsilon_{i}A_{i}\left(\tilde{q}\right)\right],$$

(33) $$J_{i}\left(q\right)=\frac{1}{2}\left[A_{i}\left(q\right)-\epsilon_{i}A_{i}\left(\tilde{q}\right)\right].$$

The conditions (29)–(31) can be transformed into [58]

(34) $$\begin{array}{cc} D(x,p) & =D(x,-p), \end{array}$$

(35) $$\begin{array}{cc} \gamma \left(x,p\right)\frac{p}{m} & =D(x,p)\frac{\partial \phi }{\partial p}, \end{array}$$

(36) $$\begin{array}{cc} \frac{p}{m}\frac{\partial \phi }{\partial x} & =\frac{dV}{dx}\frac{\partial \phi }{\partial p}, \end{array}$$

for the FP Equation (6) used in this work. The first condition (34) is fulfilled under the assumption (15). The third condition (36) holds if ϕ is a function of the energy ϕ=ϕ(E) or, equivalently, if the stationary solution $\rho_{s}$ is only energy-dependent. Furthermore, the second condition (35) can be transformed into

(37) $$\frac{\gamma (x,p)}{D(x,p)}=\frac{m}{p}\frac{\partial \phi }{\partial p}=\frac{\partial \phi }{\partial E}=-\frac{d}{dE}\ln\rho_{s},$$

which is equivalent to the previous solution (7). Therefore, the stationary solution (13) satisfies the principle of detailed balance for the FP Equation (6) under the assumptions (14) and (15). Similar conclusions can be obtained for the solution (19) of Equation (16).

For the overdamped FP Equation (23), one can derive the corresponding coefficients

(38) $$A=-\frac{1}{\gamma }\frac{dV}{dx}+\frac{\partial }{\partial x}\frac{D}{\gamma^{2}},\;B=\frac{2D}{\gamma^{2}},\;Q=A,\;J=0,$$

in comparison with Equation (27). Hence, the sufficient and necessary conditions of the detailed balance could be simplified by substituting Equation (38) into Equations (29)–(31) as follows:

(39) $$\frac{\gamma (x)}{D(x)}\frac{dV}{dx}=\frac{\partial \phi }{\partial x}\;\Rightarrow \;\frac{\gamma (x)}{D(x)}=\frac{d\phi }{dV}=-\frac{d}{dV}\ln\rho_{s},$$

which is consistent with the solution (24). Thus, the stationary solution (26) satisfies the principle of detailed balance for the overdamped FP Equation (23) under the GFDR (25).

## 4. Conclusions

The GFDRs that produce the RKDs are studied in this paper. By modeling the Brownian particle in the potential with the general two-variable Langevin Equation (3) and its associated FP Equation (6), the RKD (13) is derived as the stationary solution under the assumption of the GFDR (12). In the absence of potential and for a uniform system, the RKD reduces to the velocity distribution (19), which is the solution of the one-variable FP Equation (16) with the GFDRs (17) and (18). Moreover, the Smoluchowski Equation (23) is obtained in the overdamped limit, and we show that its stationary solution is also the RKD (26) with the GFDR (25). In addition, we prove that the principle of detailed balance holds for all the stationary solutions (13), (19), and (26), ensuring the consistency of the derived distribution with the underlying physical principles. Our results may be considered as a possible physical mechanism for explaining the RKD in plasmas.

## Figures and Tables

**Figure A1:**
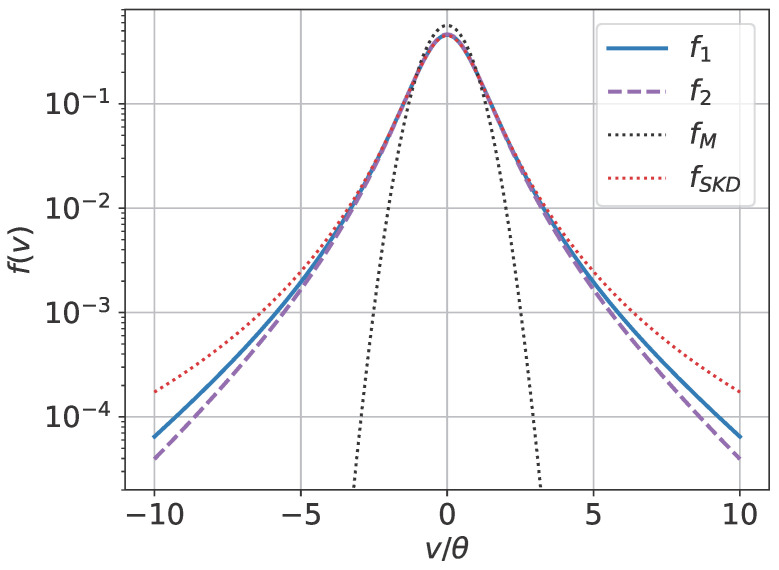
The comparison between the two kinds of one-dimensional RKDs. We denote Equation (A1) by the blue solid line, and Equation (A2) by the purple dashed line. The SKD (red dotted lines) and Maxwellian distribution (black dotted lines) are plotted as references. The parameters κ=2 and α=0.1 are set.

## Data Availability

No new data were created or analyzed in this study. Data sharing is not applicable to this article.

## Abbreviations

The following abbreviations are used in this manuscript:

SKD	Standard Kappa distribution
RKD	Regularized Kappa distirbution
SFDR	Standard fluctuation–dissipation relation
GFDR	Generalized fluctuation–dissipation relation
FP	Fokker–Planck

## Appendix A

There are two approaches to derive the one-dimensional RKD: one is to regularize the one-dimensional SKD, and the other is to integrate the three-dimensional RKD. In the absence of the potential, the first kind of RKD is

(A1) $$f_{1}=\frac{1}{\sqrt{\pi \kappa }\theta U\left(1/2,3/2-\kappa ,\alpha^{2}\kappa \right)}{\left(1+\frac{v^{2}}{\kappa \theta^{2}}\right)}^{-\kappa }\exp\left(-\alpha^{2}\frac{v^{2}}{\theta^{2}}\right),$$

and the second one is obtained by integrating Equation (2) [54]

(A2) $$f_{2}=\frac{e^{\alpha^{2}\kappa }{\left(\alpha^{2}\kappa \right)}^{\kappa }}{\sqrt{\pi \kappa }\theta U\left(3/2,3/2-\kappa ,\alpha^{2}\kappa \right)}\Gamma \left[-\kappa ,\alpha^{2}\left(\kappa +\frac{v^{2}}{\theta^{2}}\right)\right].$$

The latter RKD could be rewritten as

(A3) $$f_{2}=\frac{U(1,1-\kappa ,\alpha^{2}\kappa +\alpha^{2}v^{2}/\theta^{2})}{\sqrt{\pi \kappa }\theta U\left(3/2,3/2-\kappa ,\alpha^{2}\kappa \right)}{\left(1+\frac{v^{2}}{\kappa \theta^{2}}\right)}^{-\kappa }\exp\left(-\alpha^{2}\frac{v^{2}}{\theta^{2}}\right),$$

due to the fact that [59]

(A4) $$\Gamma \left(1-a,z\right)=e^{-z}z^{1-a}U\left(1,2-a,z\right).$$

Thus, one finds that

(A5) $$\frac{f_{2}}{f_{1}}=\frac{U(1/2,3/2-\kappa ,\alpha^{2}\kappa )}{U(3/2,3/2-\kappa ,\alpha^{2}\kappa )}U\left[1,1-\kappa ,\alpha^{2}\left(\kappa +v^{2}/\theta^{2}\right)\right].$$

Figure A1 shows the comparison of these two kinds of RKDs. In addition, the one-dimensional SKD,

(A6) $$f_{SKD,1D}=\frac{1}{\sqrt{\pi \kappa \theta^{2}}}\frac{\Gamma (\kappa )}{\Gamma (\kappa -1/2)}{\left(1+\frac{v^{2}}{\kappa \theta^{2}}\right)}^{-\kappa },$$

and the Maxwellian one, $f_{M}=\exp\left(-v^{2}/\theta^{2}\right)/\sqrt{\pi \theta^{2}}$, are plotted in this figure as references.
